# Novel and known mutations of *TGFBI,* their genotype-phenotype correlation and structural modeling in 3 Chinese families with lattice corneal dystrophy

**Published:** 2010-02-15

**Authors:** Xingwu Zhong, Suqin Chen, Weijun Huang, Jun Yang, Xiaolian Chen, Yan Zhou, Qiang Zhou, Yiming Wang

**Affiliations:** 1 Zhongshan Ophthalmic Center and State Key laboratory of ophthalmology, Sun Yat-Sen University, Guangzhou, P.R.China; 2 Department of Medical Genetics, Zhongshan Medical College, Sun Yat-Sen University, Guangzhou, P.R.China; 3 Fudan University and Chinese National Human Genome Centre, Shanghai, China

## Abstract

**Purpose:**

To report novel transforming growth factor beta-induced (*TGFBI)* mutations responsible for lattice corneal dystrophy (LCD), the associated genotype-phenotype correlation, and structural changes in the mutant proteins in three Chinese families.

**Methods:**

Three unrelated Chinese families were diagnosed as Type I LCD. Mutations in *TGFBI* were detected by sequencing all of the 17 exons and splice sites of the gene. Phenotype, including corneal erosions, and opacification in the families were compared. Structural changes of the mutant proteins were modeled. One hundred healthy volunteers were recruited as controls for sequence analysis of *TGFBI*.

**Results:**

Two novel mutations, c.(1702G>C and 1706T>A; p.Arg514Pro and Phe515Leu) in *TGFBI* were identified in Family 1. Two known hotspot mutations, c. 531C>T (p. Arg124Cys) and c.1876A>G (p.His572Arg), were revealed in Family 2 and Family 3, respectively. Sequence analysis in the 100 healthy control subjects, the unaffected members in Family 1, and evolutionary alignment showed that the novel mutations occurred in the conserved amino acids. Structural modeling revealed changes in the 2nd structure of the mutant proteins, but did not detect gross structural changes. Mutations c.(1702G>C and 1706T>A; p.Arg514Pro and Phe515Leu) and the c. 531C>T (p. Arg124Cys) were present in the corneas with sever opacification.

**Conclusions:**

The novel mutations c.(1702G>C and 1706T>A; p.Arg514Pro and Phe515Leu), c. 531C>T (p. Arg124Cys), c.1876A>G (p.His572Arg) in *TGFBI* were responsible for LCD in the 3 families. Mutations c.(1702G>C and 1706T>A) (p.Arg514Pro and Phe515Leu) and the c. 531C>T (p. Arg124Cys) were associated with more severe LCD phenotypes in the families. These results provide more data for molecular diagnosis and prognosis of the disease.

## Introduction

Lattice corneal dystrophy (LCD) is an inherited disease characterized by the accumulation of amyloid materials that form refractile lines and white dots in the corneal stroma. Genetically, this disease is classified into five distinct subtypes, type I, II, III, and IIIA, and IV [1–3]. Patients usually present with ocular pain and recurrent corneal erosions in the first or second decade of life. Corneal opacification and blindness could eventually occur, requiring keratoplasty to restore sight. Type I LCD is the most common subtype and the disease usually progresses slowly [4]. These clinical findings are the characteristics of LCD, and genetic classification does not really reflect them; therefore, a classification that includes genetics and phenotype appears to be more practical. 

Genetically, transforming growth factor beta-induced (*TGFBI* Entrez Gene ID: 7045) is the gene underlying most incidences of LCD. The dominant mode of inheritance has been recognized as the pattern of transmission [5,6], although homozygous mutations, for the severe forms of corneal dystrophy, and compound heterozygous mutations have been reported [7]. To date more than 30 mutations of *TGFBI* are responsible for corneal dystrophy, with various clinical subtypes being identified [7]. However, no structural modeling of the mutant proteins has been reported. In this paper, we report 2 novel mutations of *TGFBI* identified in one Chinese family, two known heterozygous mutations in the other two families, and genotype-phenotype correlation and structural analysis of the four mutant proteins in the three Chinese families with LCD.

## Methods

### Patients and control subjects

**Figure 1:**
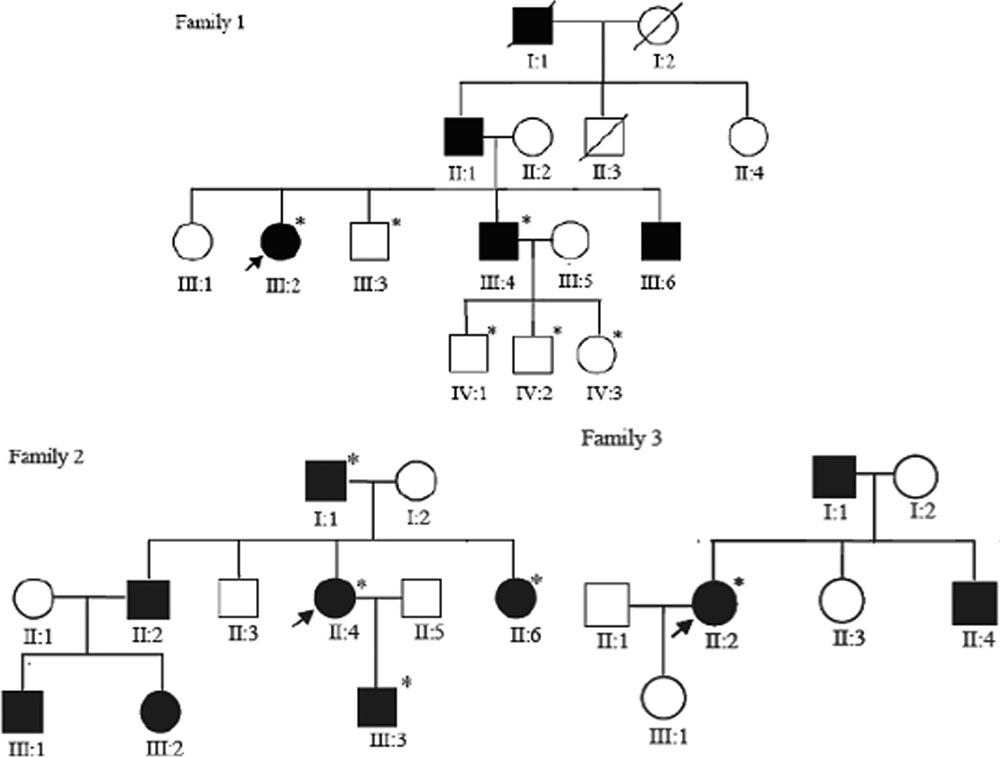
Pedigrees of Family 1, 2, and 3. The closed symbols represent individuals affected by the LCD and the open symbols represent those who are unaffected. Arrowheads denote probands. Asterisks point to members analyzed in the study.

Five members from Family 1, four members from Family 2, one member from Family 3, and one hundred healthy volunteers (Han ethnicity) in clinic of Zhongshan Ophthalmic Center were recruited for this study. This study was approved by the Review Board of Sun Yat-Sen University. The principles outlined in the Declaration of Helsinki, such as participant safety, clinical trial registration, post-study access, usage of data and human tissues, compensating participants with research-related injury, were followed. Pedigrees of the three families were constructed (Figure 1). Among the three families, Family 1 and Family 2 came from Guangdong province, in the south of China, and Family 3 came from Sichuan province, in Western China. All three families are of Han ethnicity. Owing to the availability of samples, ten members of the families were analyzed (III:2, III:3, III:4, IV:2, IV:3 in Family 1; I:1, II:4, II:6, III:3 in Family 2; and II:2 in Family 3; Figure 1). No consanguinity between the families was found in the family histories. Visual acuity, slit lamp microscopy, and funduscopic examinations were performed by ophthalmologists (X.Z. and J.Y.). Blood samples were collected for DNA isolation. All the individuals included in the study underwent clinical examination before the molecular investigation. Systemic amyloidosis was excluded in all of the affected family members. One hundred healthy volunteers (Han ethnicity) were recruited as controls for sequence analysis of *TGFBI*. These control subjects were free of corneal opacity and epithelial defect, which was confirmed by slit-lamp microscopy.

### Mutational analysis

Genomic DNA from the peripheral blood of the available family members was extracted with a QiaAmp Kit (Qiagen). All of the 17 exons and flanking intronic sequences of *TGFBI* (Entrez Gene ID: 7045) were amplified by polymerase chain reaction (primer sequences and PCR conditions are available on request). The products were purified with a Pre-Sequencing Kit (USB, Cleveland, OH) and sequenced in both directions using a BigDye Terminator v3.1 Cycle Sequencing Kit and an ABI 3100 Genetic Analyzer (Applied Biosystems, Foster City, CA). The results were compared with the sequence retrieved from the UCSC Genome Browser. The HGVS guidelines for describing sequence variations and numbering were used. Haplotypes were constructed using the *TGFBI* locus markers D5S816, D5S393, and the c.1702G>C and c.1706T>A variants in Family 1.

### Evolutionary comparisons of the TGFBI ortholog

The amino acid sequences of the TGFBI orthologs of chimpanzees (ENSPTRG00000017264), dogs (ENSCAFG00000001091), rats (ENSRNOG00000012216), mice (ENSMUSG00000035493), and chicks (ENSGALG00000006319) were retrieved from the Ensembl Genome Browser and compared with the human TGFBI amino acid sequences (AAA61163.1).

### Structural modeling of the wild and mutant proteins

The SWISS MODEL was used to model the structure of the wild type protein and the four mutant proteins. The 3D protein models were viewed using RasMol. The secondary structures were predicted by PredictProtein, NNPREDICT, and PROF to obtain a comprehensive understanding of the effect of the mutations found in the three families.

## Results

### Clinical findings

All of the affected family members examined showed anterior refractile corneal stromal deposition characterized by several branching and nonbranching lattice figures resembling pipe stems in both eyes. These patients also had delicate, filamentous, discrete, short, and irregularly shaped stromal deposition, along with corneal haze.

**Figure 2:**
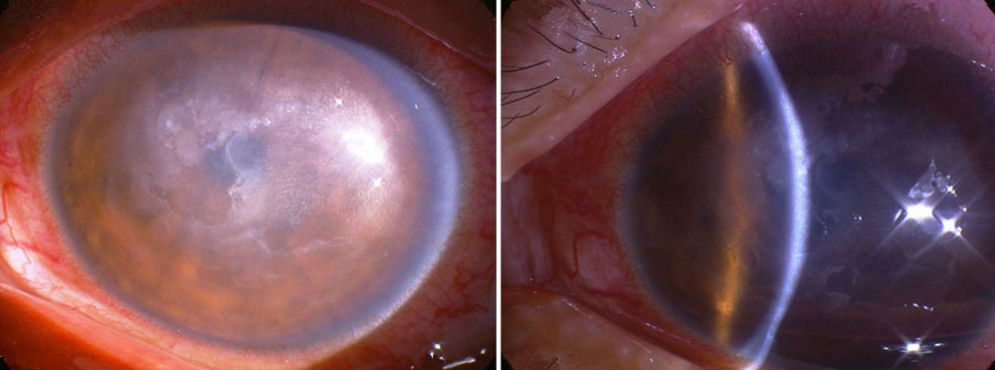
Thick lattice lines and a subepithelial clump resulting in reduced visual acuity as low as 20/200 in the 36-year-old man in Family 1 (III-4).

The onset of all the affected individuals in Family 1 was in the second decade of life. Ocular pain, recurrent corneal erosions, and corneal opacification were the frequent complaints and clinical signs of LCD. These symptoms progressed more rapidly in relation to the other families. Penetrating keratoplasty was necessary in the patients’ late 20s (Figure 2).

**Figure 3:**
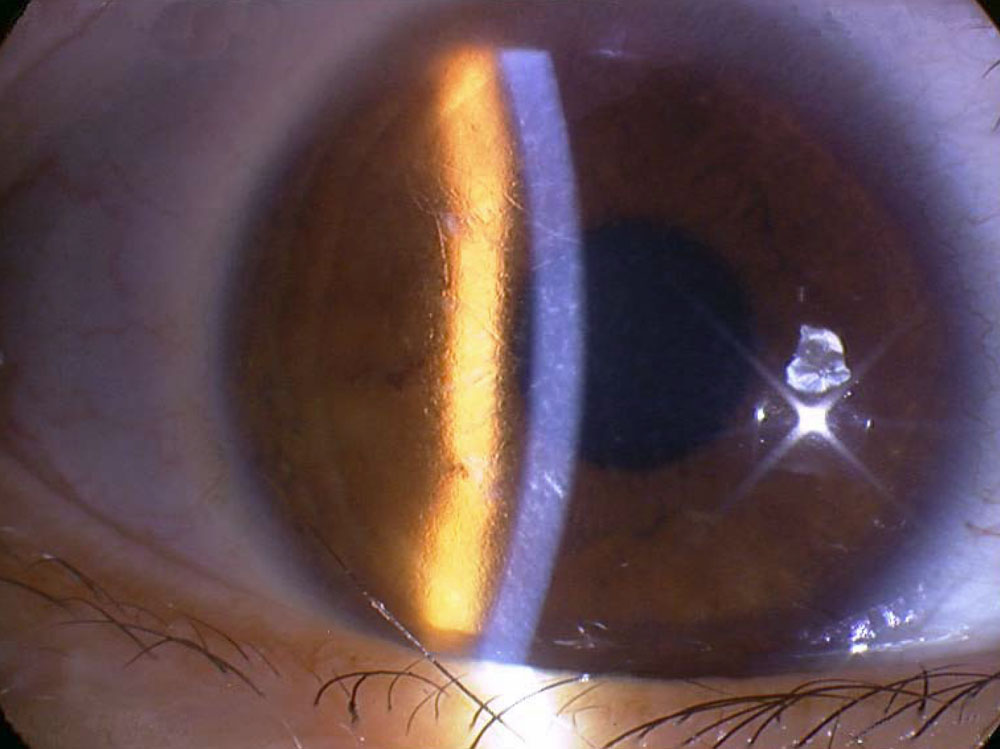
The affected individual of Family 2 (II-4) at 28 years of age; a thin lattice of deposition in the stromal layer associated with subepithelial smaller opaque spots is shown.

**Figure 4:**
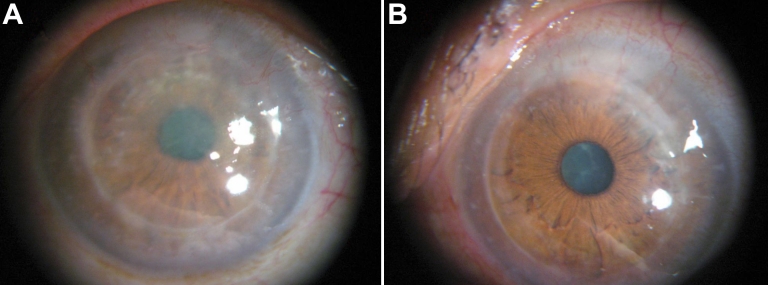
Clinical photographs of corneas in one affected member (I-1) in Family 2 at 63 years of age. The right eye (**A**) contained opacifications from presumed recurrent disease 20 years after a penetrating keratoplasty. The left eye (**B**) showed a corneal implant that was transparent after a penetrating keratoplasty 10 years ago. This eye compounded with the primary cataract and the corrected visual acuity with pinhole is 20/32.

The proband (II-4) patient in Family 2 had visual loss in both eyes when she was approximately 23 years old. On examination, subepithelial punctiform corneal erosions were present in both eyes (Figure 3). In 1985, her father (I-1) had penetrating keratoplasty for the first time on his right eye at age 41. One year later, the corneal implant was rejected. He had penetrating keratoplasty twice in both eyes one year later. Due to recurrence of the primary disease, he had another penetrating keratoplasty on his left eye ten years later (Figure 4). Unfortunately, his histopathological record could not be retrieved. Her son (III-3) experienced a foreign body sensation in both eyes, however, no abnormalities were found in his corneas.

Family 2 was a branch of a large Chinese family of Han ethnicity living in southern China. The family members with LCD encompass five generations. Some members of this family were living overseas. We learned from the proband (II-4) that, like her, some members of the family had analogous symptoms in their teenage years, and some members had undergone penetrating keratoplasty around age 40. However, we cannot find their medical records. We have invited the other members of this family to our study, but have yet to hear from them.

**Figure 5:**
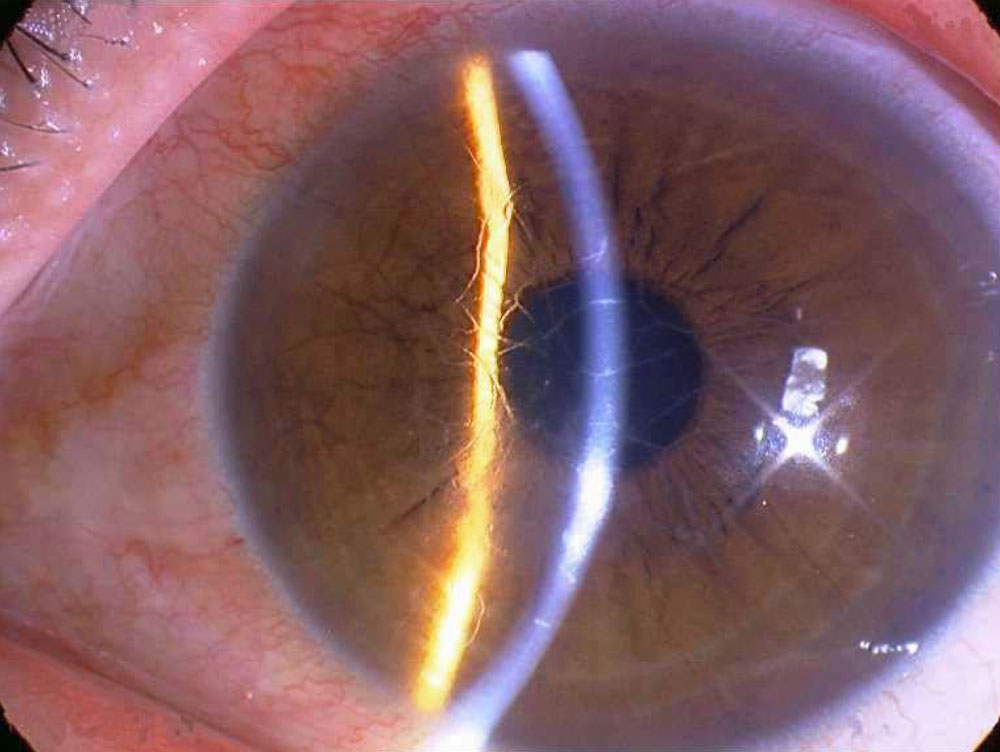
The proband of Family 3(II-2) at the 38 years of age with thin linear irregular branching refractile lines and white dots deposition in the subepithelial and stromal layers. Visual impairment was not obvious.

The proband (II-2) from Family 3 displayed thin linear branching deposition in the subepithelial and stromal layers in both eyes that first appeared during adulthood. The disease progression in this case was slow. The patient also had recurrence of corneal erosions. No visual impairment was found during examinations when he/she was 38 years old (Figure 5).

### Genetic findings

**Figure 6:**
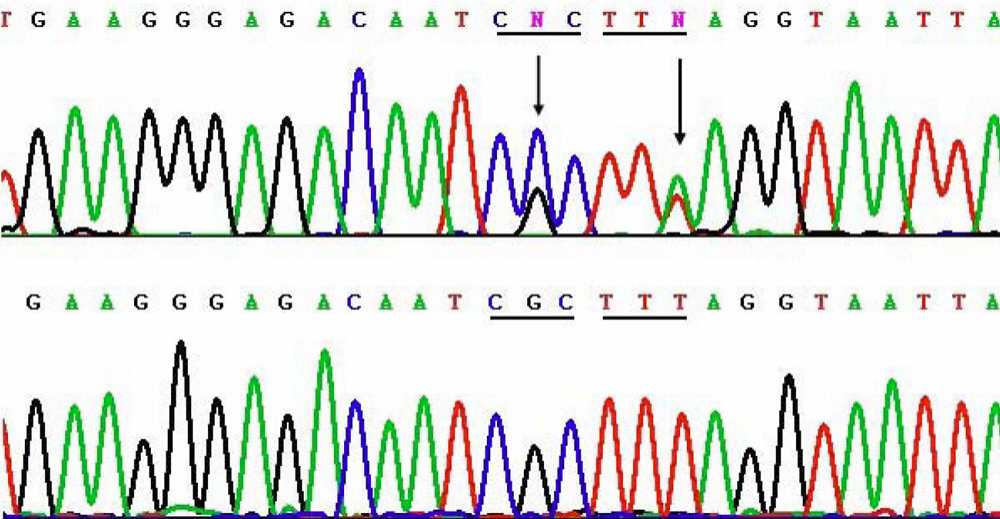
Sequencing results of the two novel mutations c.1702G>C and 1706T>A (p.Arg514Pro and Phe515Leu) of *TGFBI* in Family 1, and their corresponding normal sequences. Arrows indicate the mutation positions. Underlines highlight the codons that contain the mutations.

**Figure 7:**
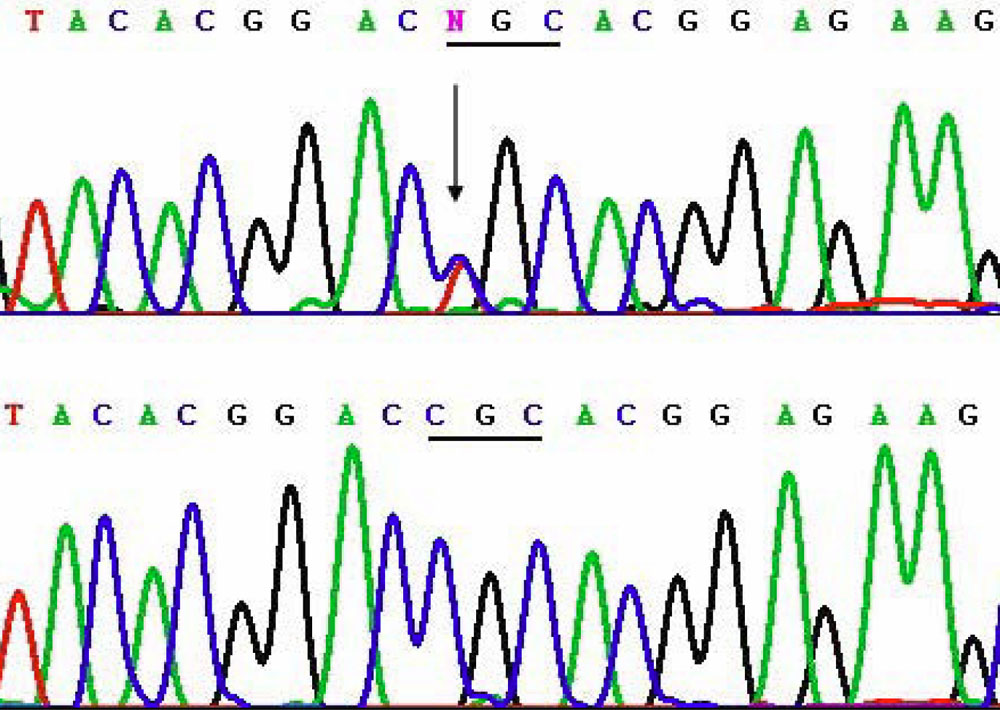
Sequencing results of the heterozygote mutation c. 531C>T (p. Arg124Cys) of *TGFBI* in Family 2, and their corresponding normal sequences. Arrows indicate the mutation positions. Underlines highlight the codons that contain the mutations.

**Figure 8:**
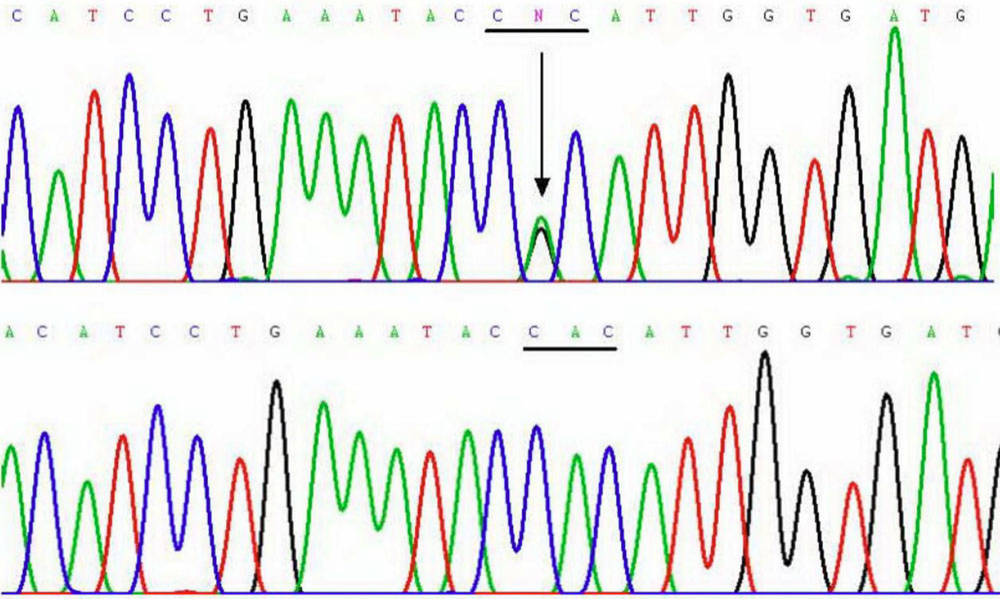
Sequencing results of the heterozygote mutations c.1876A>G (p.His572Arg) of *TGFBI* in Family 3, and their corresponding normal sequences. Arrows indicate the mutation positions. Underlines highlight the codons that contain the mutations.

Pedigree analysis showed an autosomal dominant inheritance pattern in all three families. In Family 1, two novel mutations, c.1702G>C and 1706T>A (p.Arg514Pro and Phe515Leu), in exon 11 of *TGFBI* (Figure 6) were identified in two of the patients (III-2 and III-4) examined, but were absent in the 4 unaffected members available for the analysis (III-3, IV-1, IV-2, and IV-3). In Family 2, a known hotspot mutation, c. 531C>T (p. Arg124Cys), in exon 4 was detected (Figure 7). A recently reported mutation [8], c.1876A>G (p.His572Arg), in exon 13 was identified in the proband of Family 3 (Figure 8).

**Figure 9:**
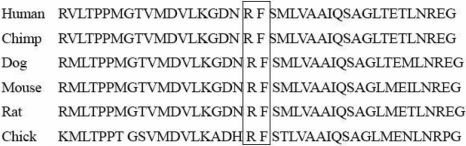
Alignment of partial amino acid (lower) sequences of *TGFBI* with 5 of its orthologs. The boxes indicate arginine at 514 and phenylalanine at 515 in the human protein sequence. These were conserved across these species.

Sequence analysis of *TGFBI* in the 100 healthy control subjects showed conserved c.1702G and c.1706T, suggesting that the c.1702G>C and 1706T>A substitutions were not polymorphisms. Owing to the lack of family members, haplotype analyses using the *TGFBI* locus markers D5S816 and D5S393 and the c.1702G>C and 1706T>A variants could not conclusively identify parental origin of the two mutations. Alignment of the amino acid sequences of TGFBI displayed a high conservation of p.Arg514Pro and Phe515Leu in the chimpanzee, cow, dog, mouse, rat, chick, and *Xenopus* orthologs (Figure 9).

### Structural modeling

Prediction with the SWISS MODEL indicated that all the mutations occurred in the alpha-helix region, which, predictively, would induce local secondary structure changes, although no gross structure modification could be detected. In Family 1, the mutation p.Arg514Pro is predicted to shorten the helix and induce the formation of a downstream turn structure; whereas the p.Phe515Leu mutation could predictably elongate the helix. The combined effects of these two mutations conformed to the result of p.Arg514Pro.

## Discussion

In the present study, the affected individuals of Family 1 and Family 2 had symptoms in the first decade of life; therefore, penetrating keratoplasty is indicated in their late 20s. This indicates that mutations c.(1702G>C and 1706T>A) (p.Arg514Pro and Phe515Leu) and c. 531C>T (p. Arg124Cys) have more phenotypic effects. The mutation c.1876A>G (p.His572Arg) is also identified in Family 3. The proband of Family 3 has thin linear branching deposits in the subepithelial and stromal layers in both eyes, with the deposition detected in adulthood. Visual impairment was not obvious in his/her 20s. The same mutation has been reported in later onset Type I LCD [9] from a Thai family. Consistently, the average age of onset of symptoms of the affected individuals in the Thai family was 28.6±8.1 years (range: 20–50). In addition, the p.His572del is associated with a unilateral, late-onset variant form of LCD in a 63-year-old man with decreased vision in the affected eye [8]. This indicates that c.1876A>G may be particularly associated with the relatively late onset of LCD and a less severe phenotype.

Predominantly expressed by the corneal epithelium, TGFBI is believed to be an adhesion protein secreted into the extracellular matrix and bound to Type I, II, and IV collagens responsible for the structure of microfibrils and cell surface. *TGFBI* is also a major gene responsible for most LCD cases. To date, more than 30 mutations in *TGFBI* have been reported to cause dominantly inherited corneal disorders [7], although homozygous mutations are also reported in severe forms of corneal dystrophy patients. Moreover, most mutations reported from present or previous studies are single base pairs of substitutions affecting one of the alleles in the locus. Compound heterozygous mutations affecting both alleles are rare. Dighiero and colleagues [10] suggest that both p.Arg124Leu and p.Thr125_Glu126del compound heterozygous mutations are responsible for the phenotype of the family with granular corneal dystrophy. The compound heterozygous mutations p.Arg124Cys coupled with p.Gly470X (a nonsense mutation) have been identified in the proband of one family [11]. However, the proband’s daughter who carries the heterozygous mutation p.Gly470X alone was unaffected. This raises the question as to whether the p.Gly470X in the compound heterozygous mutations could be pathogenic. The authors speculated that the p.Gly470X nonsense mutation might be nonpathogenic or have a very low penetrance. The third compound heterozygous mutations, p.Ala546Asp and p.Pro551Gln, have been reported in two non-consanguineous African-American families [12].

In an attempt to distinguish the pathogenic mutations from polymorphism in the compound mutations [12], haplotype analysis of the *TGFBI* locus and sequenced samples from 125 healthy controls show no common haplotype between the affected and unaffected family members and no mutations in the control subjects. In this present study, 2 novel mutations, c.1702G>C and 1706T>A (p.Arg514Pro and Phe515Leu), are identified in Family 1. The two substituted base pairs are only 4bp away from each other, and affect the consecutive amino acids in the polypeptide. This also raises the question whether the two mutations are located in one allele consecutively, or in both alleles separately (one in each of the alleles). These questions are unable to be answered due to lack of sufficient family samples for the analysis. However, the pedigree looks like a dominant inheritance in the family, as the spouses (I:2, II:2) of the mutant affected members (I-1, II-1) are unaffected. Regardless of whether the two mutations occur in one or both alleles, the pathogenic roles of the mutations are likely associated with severity in the phenotype of LCD, as they are absent in the unaffected family members and controls subjects. The 2nd structural changes of the mutant protein by either one or both of the mutations also provide support of their pathogenic roles. This is also consistent with the phenotype severity in the family, as it has an earlier age of onset, relatively faster progression, and requires earlier penetrating keratoplasty compared to Family 3.
